# Effect of Magnesium on Dentinogenesis of Human Dental Pulp Cells

**DOI:** 10.1155/2021/6567455

**Published:** 2021-11-18

**Authors:** Rania M. Salem, Chang Zhang, Laisheng Chou

**Affiliations:** ^1^Department of Restorative Sciences & Biomaterials, Goldman School of Dental Medicine, Boston University, Boston, MA 02118, USA; ^2^Department of Endodontics, Goldman School of Dental Medicine, Boston University, Boston, MA 02118, USA

## Abstract

Introducing therapeutic ions into pulp capping materials has been considered a new approach for enhancing regeneration of dental tissues. However, no studies have been reported on its dentinogenic effects on human dental pulp cells (HDPCs). This study was designed to investigate the effects of magnesium (Mg^2+^) on cell attachment efficiency, proliferation, differentiation, and mineralization of HDPCs. HDPCs were cultured with 0.5 mM, 1 mM, 2 mM, 4 mM, and 8 mM concentrations of supplemental Mg^2+^ and 0 mM (control). Cell attachment was measured at 4, 8, 12, 16, and 20 hours. Cell proliferation rate was evaluated at 3, 7, 10, 14, and 21 days. Crystal violet staining was used to determine cell attachment and proliferation rate. Alkaline phosphatase (ALP) activity was assessed using the fluorometric assay at 7, 10, and 14 days. Mineralization of cultures was measured by Alizarin red staining. Statistical analysis was done using multiway analysis of variance (multiway ANOVA) with Wilks' lambda test. Higher cell attachment was shown with 0.5 mM and 1 mM at 16 hours compared to control (*P* < 0.0001). Cells with 0.5 mM and 1 mM supplemental Mg^2+^ showed significantly higher proliferation rates than control at 7, 10, 14, and 21 days (*P* < 0.0001). However, cell proliferation rates decreased significantly with 4 mM and 8 mM supplemental Mg^2+^ at 14 and 21 days (*P* < 0.0001). Significantly higher levels of ALP activity and mineralization were observed in 0.5 mM, 1 mM, and 2 mM supplemental Mg^2+^ at 10 and 14 days (*P* < 0.0001). However, 8 mM supplemental Mg^2+^ showed lower ALP activity compared to control at 14 days (*P* < 0.0001), while 4 mM and 8 mM supplemental Mg^2+^showed less mineralization compared to control (*P* < 0.0001). The study indicated that the optimal (0.5–2 mM) supplemental Mg^2+^ concentrations significantly upregulated HDPCs by enhancing cell attachment, proliferation rate, ALP activity, and mineralization. Magnesium-containing biomaterials could be considered for a future novel dental pulp-capping additive in regenerative endodontics.

## 1. Introduction

More than two-thirds of the global population suffer from tooth decay, which results in cavities with various levels of lesion severity [1–3]. Although surface decay can usually be treated and arrested with a filling, a tooth with deep decay or one that has been severely injured in an accident, or the trauma of recurrent dental work may become unhealthy, inflamed, or infected. Left untreated, an infection may spread to the surrounding tissues [4]. The currently available treatment scenarios in such serious conditions are either vital pulp therapy if the pulp is still vital or root canal treatment if the pulp is irreversibly inflamed. Vital pulp therapy aims to induce dentinal bridge formation with scarred tissue to maintain pulp integrity and function. This mechanism of tissue repair is based on replacing damaged odontoblasts by newly regenerated populations of odontoblast-like cells derived from pulp stem cells in the healthy portion of the pulp [5, 6].

To date, calcium hydroxide (CH), mineral trioxide aggregate (MTA), and tricalcium silicate cement (Biodentine) are the pulp-capping biomaterials of choice mostly used in clinics [7]. Historically, calcium hydroxide (CH) has been considered the gold standard in recent decades [8]. However, CH has many disadvantages, such as high solubility and multiple tunnel defects in the induced dentin bridge [9], which allow the pulp to become infected or necrotic over time [10, 11]. The first calcium silicate cement-based material, mineral trioxide aggregate (MTA), is used in direct and indirect pulp capping in primary and permanent teeth [12, 13]. MTA has been reported superior to calcium hydroxide for pulp capping of mechanically exposed human teeth [14–16]. However, MTA exhibits many drawbacks such as difficult handling, long setting time [17], induction of tooth discoloration, and incompatibility with other dental materials when layered [18]. Therefore, a new formulation named Biodentine (Septodont, Saint Maur, France) has been launched [19, 20], with physicomechanical properties superior to those of MTA and similar to those of dentin, easier handling and shorter setting time than MTA [21], and positive effects of biodentine stimulating cell differentiation and promoting mineralization in human dental pulp cells [22, 23]. However, the main drawback is its water-based chemistry, and thus, poor bonding as the bond is mainly micromechanical to the overlying resin restoration [24]. Regardless of which materials used, however, there are notable limitations in the current biomaterials‐based strategies used for pulp capping and dentin regeneration. Some of the most significant limitations are the severe inflammatory reactions induced by the synthetic-capping materials that can cause failure. Besides, formation of the dentinal bridge can occur within the teeth with irreversible inflammation, which requires comprehensive retreatment [25, 26]. These limitations mainly derive from the fact that current biomaterials used in the clinics for healing dentin‐pulp tissues lack specific temporal and spatial control over biologic signaling required for progenitor cells' homing and differentiation to eventually fully restore structural and functional characteristics of the tissue. Moreover, the mechanistic understanding of the regenerative outcomes attained using current pulp-capping biomaterials is for the most part missing. Finally, current biomaterials are notably limited to control infection and inflammation for promoting reparative tissue formation [27].

Critical need exists to develop a novel therapy that induces wound healing and dentinogenesis similar to the natural process. The development of pulp-capping agents has been instrumental in promoting reparative dentin formation. Incorporation of Mg^2+^ into pulp-capping materials allowing the release of therapeutic Mg^2+^ions has been considered as a new approach for enhancing regeneration of dental tissues. Therapeutic ions induce cellular signaling, triggering resident pulp cells to differentiate into odontoblast-like cells, stimulation of dentin matrix secretion, and formation of tertiary dentin. Mg^2+^acts as an intracellular second messenger connecting cell-surface receptor induction and cytosolic effectors [28]. Mg^2+^ is the fourth most abundant cation in the human body and is critical for ATP-dependent phosphorylation of DNA, RNA, and enzymes. Mg^2+^ is present (0.5%) in the tooth enamel outer layer, and the average magnesium concentration is 1% (w/w) in dentin [29–31]. Mg^2+^ is involved in the biomineralization of bones and teeth and directly affects crystallization and pattern generation of the inorganic mineral phase [31–33]. Emerging evidence supports a notion that Mg^2+^ plays indispensable bioactive roles and not only induces bone formation and matrix mineralization but also in transduction of the intracellular signaling pathway that are involved in maintaining and regulating normal biological processes [34]. Mutation of Mg^2+^ transporters CNNM4 and TRPM7 resulted in mineralization defects of dentin, indicating that a disrupted Mg^2+^ transport was involved in the development of the dental abnormalities. Animals fed on low Mg^2+^ diets show deficient dentin and enamel mineralization [35].

This is the first report exploring Mg^2+^ application and its dentinogenic effects on normal human dental pulp cells. The mechanism behind magnesium relationship to cell behavior is intriguing largely unexplored. Thus, our objective in this unique study was to investigate the effect of different concentrations of supplemented Mg^2+^ on human dental pulp cells, looking specifically at cellular proliferation, differentiation, and mineralization which may contribute to a better understanding of the influence of magnesium on pulp regeneration. Its potential application in clinical practice as a promising material for clinical pulp regenerative therapy is to establish an optimal concentration that could ultimately be used to develop a new pulp-capping material.

## 2. Materials and Methods

### 2.1. Magnesium Chloride Preparation

Magnesium chloride hexahydrate (Fisher Chemical, USA) was dissolved in deionized water, and five stock solutions were prepared at concentrations of 5 mM, 10 mM, 20 mM, 40 mM, and 80 mM, respectively. Each concentration was subsequently filtered under sterile condition in the biological hood.

### 2.2. Human Dental Pulp Cells (HDPCs) Culture

This study was approved by Boston University IRB Committee. Human dental pulp explants were collected from young and systemically heathy patients between the age group of 15 and 25 years, requiring third molar or orthodontic premolar extractions. Patients were given the informed consent before the extraction procedure at the oral surgery clinic at Boston University. Human dental pulp cells (HDPCs) were isolated following a previously published protocol with modifications [36]. Teeth were sectioned with a #7 chandler bibevel bone chisel until the pulp tissue in the pulp chamber was exposed. The pulp pieces were removed with sterile instruments and placed immediately into a 12.5 cm^2^ culture flask (Thermo Fisher Scientific, USA). Culture medium consisted of 10% fetal bovine serum (FBS) (R&D Systems), 1X penicillin antibiotic (100 U/mL), 1X streptomycin (100 *μ*g/mL) (Gibco), and amphotericin B antifungal (0.25 *μ*g/ml) in Eagle's basal medium (BME) (Gibco). All tissues were maintained at 37°C, in a standard 5% CO_2_ incubator (Thermo Fisher Scientific, USA) and saturated humidity and cultured up to the second passage. Growth media was changed every 72 hours. Nearly, confluent cells were trypsinized with 0.05% trypsin (Gibco). Resuspended cells were then aspirated and collected in a sterile 15 mL disposable tube placed in the TJ-6 Beckman centrifuge at 1000 rpm for 5 minutes. After centrifugation, a pellet of cells was formed. The cells were then counted using a hemocytometer. Characterization of dentinogenic phenotype of the cells was confirmed by expression of dentinogenic markers induced by vit D_3_ stimulation. Human dental pulp cells were transferred to 24-well plates (Thermo Fisher Scientific, USA) and grown in the culture medium supplemented with 0 mM (control), 0.5 mM, 1 mM, 2 mM, 4 mM, and 8 mM supplemental Mg^2+^ concentrations, respectively. For differentiation and mineralization studies, growth media were replaced with preinductive dentinogenic media at the following time intervals: 4, 7, and 11 days. Dentinogenic media consist of the following: 10% charcoal-stripped fetal bovine serum (FBS) (Life Technologies), 100 IU/ml penicillin (Gibco), 100 *μ*g/m streptomycin (Gibco), 10^−8^ M menadione (Sigma-Aldrich), 10 mM *β*-glycerophosphate (Sigma-Aldrich), 0.05 mg/mL L-ascorbic acid (Sigma-Aldrich), and 2 mM L-glutamine (Gibco). The next day, cells were cultured in preinductive dentinogenic media with the addition of 10 nM vitamin D_3_ (172 g/mol) (Sigma) for two additional days. Supernatant fluid was collected on days 7, 10, and 14. Alkaline phosphatase (ALP) activity was measured in the collected supernatants. The remaining fixed cells on the culture plates were used to perform the mineralization assay.

### 2.3. Determination of Human Dental Pulp Cells (HDPCs) Attachment Efficiency and Proliferation Assays

Two hundred thousand (2 × 10^5^) normal human dental pulp cells were seeded in 24-well plates containing 1 mL media with 0 mM, 0.5 mM, 1 mM, 2 mM, 4 mM, and 8 mM supplemental Mg^2+^ concentrations for 16 hours. Each condition was performed in six replicas. Supernatants were collected in 1.5 mL disposable tubes. Preexperiments were performed to determine the baseline of cell attachment at 16 hours. After 16 hours, the medium was discarded, and the wells were washed 3 times with phosphate-buffered saline (PBS) (Gibco). Thereafter, the cells were fixed by adding 500 *μ*L of 10% neutral buffered formalin (Sigma) for 1 hour at room temperature. The cells were then stained by adding 500 *μ*L of 0.2% crystal violet stain (Sigma-Aldrich) for another hour. Afterwards, all wells were washed 3 times using PBS to remove any unbound stains. The optical density of the stained cells was measured by the spectrophotometer (TECAN, Infinite 200 Pro) at wavelength 590 nm. The optical density is directly proportional to the cell numbers. Cell attachment efficiency was quantified through direct cell counts and normalized to the initial cell seeding density. Cell proliferation was monitored at the following time points: 16 hours, 3 days, 7 days, 10 days, 14 days, and 21 days. Three thousand normal human dental pulp cells were seeded in 24-well plates containing 1 mL of growth medium for the control group (0 mM) and various concentrations of supplemental Mg^2+^ 0.5 mM, 1 mM, 2 mM, 4 mM, and 8 mM, respectively. Each condition has been repeated in six replicas. The culture plates were incubated under 37°C, 5% CO_2_, and growth media were changed every 3 days. At each predetermined time point, 0.2% crystal violate dye was used to stain the attached cells in the 24-well plates. Absorbance of crystal violet was measured using the spectrophotometer at 590 nm wavelength. The optical densities at each point of time were compared to the optical density at 16 hours as a baseline to determine the proliferation rates.

### 2.4. Measurement of Alkaline Phosphatase (ALP) Differentiation Marker Activity

An alkaline phosphatase (ALP) fluorometric assay kit (Abcam) was used to measure alkaline phosphatase activity in the cell culture supernatants. Supernatants at days 7, 10, and 14 were utilized. ALP activity was measured according to the manufacturer's instructions. 100 *μ*L (10x diluted) culture supernatants were incubated with 20 *μ*L of the nonfluorescent 4- methylumbelliferone phosphate disodium salt (MUP substrate) in a 96-well black plate with clear bottom (Thermo Scientific); MUP was converted into fluorescent 4-methylumbelliferone (4-MU) when dephospholated by ALP. The plate was incubated for 30 minutes at 25°C protected from light. The reaction was then terminated by 20 *μ*L stop solution, which was added to all wells, with the exception of the blank control, and the plate was gently shaken. The emission of the fluorescent 4-MU was measured at 440 nm by excitation at 360 nm on the spectrophotometer. ALP activities were calculated by a standard curve and normalized to ALP activity on a per million cell base formula.

### 2.5. Detection of Human Dental Pulp Cells (HDPCs) Mineralization

Mineralization was examined through accumulation of calcium deposition using Alizarin red S staining. The attached cells in the 24-well plates at days 7, 10, and 14 were used. The crystal violet stain from the plates was removed by adding Triton-X 1% (v/v) (900 *μ*L). The 24-well plates were placed on a shaker for 30 min at room temperature. The plates were washed cautiously four times with 1 ml deionized water to the point where colorless. Later, 1 mL of 40 mM Alizarin red S (Sigma) pH 4.3 solution was added to the plates for calcium staining. Cells were incubated at room temperature in the dark for 45 min. Then, Alizarin Red S staining was carefully aspirated, and the plates were washed four times with 1 ml distilled water until clear. Finally, absorbance was measured by the spectrophotometer at a wavelength of 405 nm. Each condition was normalized to a per million cell base formula.

### 2.6. Statistical Analysis

All experiments were performed in six replicates and repeated three times. Data are presented in means and standard deviations. The means and standard deviations (SD) of human dental pulp cell attachment efficiency and proliferation rates at 16 hours and 3, 7, 10, 14, and 21 days were calculated, in addition to levels of osteogenic differentiation marker (alkaline phosphatase) and mineralization at 7, 10, and 14 days. Differentiation and mineralization data were normalized on a per million cells basis at 7, 10, and 14 days. Statistical analysis was performed using software JMP Pro 13 (ver. 13.1.0). Multiway analysis of variance (multiway ANOVA**)** with Wilks' lambda test is used for statistical analysis between the groups. Differences at *P* ≤ 0.05 were considered statistically significant.

## 3. Results

### 3.1. Effect of Supplemental Mg^2+^ on Cell Attachment Efficiency of HDPCs at Various Concentrations

**Figure 1:**
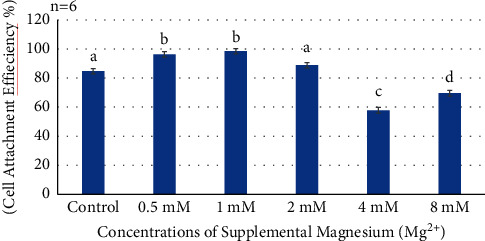
Histogram showing cell attachment efficiency at 16 hours of all supplemental Mg^2+^ concentrations. Note: normal human dental pulp cells were cultured for 16 hours with media containing supplemental Mg^2+^ concentrations 0.5 mM, 1 mM, 2 mM, 4 mM, and 8 mM and 0 mM as the control group. The data are presented as means of six replicates with error bars indicating the standard deviation. Groups labeled with different letters differ statistically as compared to the control group and other study groups (*P* < 0.0001).

Adherence of HDPCs occurred within a few hours after cells were seeded. Cell attachments on the tissue culture plates were significantly greater in Mg^2+^ concentrations among certain groups (*P* ≤ 0.0001) after 16 hours (Figure 1). The data showed a significant increase in cell attachment for the 0.5 mM and 1 mM supplemental Mg^2+^concentrations compared to the negative control. Meanwhile, there was no significant difference in cell attachment between the 2 mm Mg^2+^ concentration compared to the negative control. However, for the 4 mM and 8 mM Mg^2+^concentrations, cell attachment was significantly lower than that of the negative control (*P* < 0.0001) (Figure 1).

### 3.2. Effect of Mg^2+^ on Proliferation Rate of HDPCs at Various Concentrations

**Figure 2:**
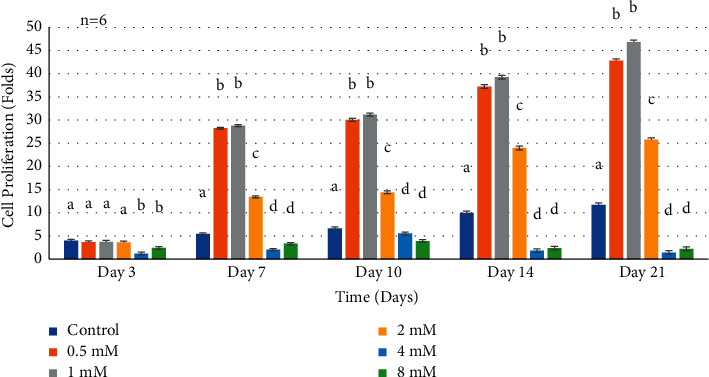
Histogram showing proliferation rates of normal human dental pulp cells in media with supplemental Mg^2+^ concentrations. Note: normal human dental pulp cells were cultured with media containing supplemental Mg^2+^concentrations 0.5 mM, 1 mM, 2 mM, 4 mM, and 8 mM and 0 mM as the control group for time periods of 3, 7, 10, 14, and 21 days. Folds were calculated by dividing the cell numbers at each time interval by the seeded cell number for each condition. Cell number was calculated by using optical density measurement results dividing the constant number (per million cells' optical density). The data are presented as means of six replicates with error bars indicating the standard deviation. Groups labeled with different letters differ statistically as compared to the control group and other study groups (*P* < 0.0001).

The proliferation rate of HDPCs at both 7 and 10 days showed a significant cell number increase in supplemental Mg^2+^ concentration groups 0.5 mM, 1 mM, and 2 mM compared to the negative control (*P* < 0.0001) (Figure 2). However, the 4 mM and 8 mM concentrations behaved similar to the control cells grown on culture media without supplements. Notably, higher supplemental Mg^2+^ concentrations resulted in a significant downregulation as shown in Figure 2. Wilks' lambda interaction *P* value for supplemental Mg^2+^ concentrations and their proliferation rate at 7 and 10 days showed a statistically significant value (*P* < 0.0001). At 14 days, the supplemental Mg^2+^ concentrations 0.5 mM, 1 mM, and 2 mM showed enhanced proliferation compared to the negative control (*P* < 0.0001). While, the 4 mM and 8 mM tested concentrations containing higher Mg^2+^significantly showed decreased growth (*P* < 0.0001). Similarly, Wilks' lambda interaction *P* value for these concentrations at 14 days showed a highly significant value (*P* < 0.0001). At 21 days, the low Mg^2+^ concentrations 0.5 mM, 1 mM, and 2 mM showed a significant increased proliferation rate (*P* < 0.001). However, the higher Mg^2+^ 4 mM and 8 mM concentrations significantly reduced proliferation (*P* < 0.0001). Wilks' lambda interaction *P* value for these concentrations at 21 days was highly significant (*P* < 0.0001).

### 3.3. Effect of Mg^2+^ on Proliferation Rate of HDPCs at Various Concentrations (after Addition of Preinductive-Dentinogenic Media)

**Figure 3:**
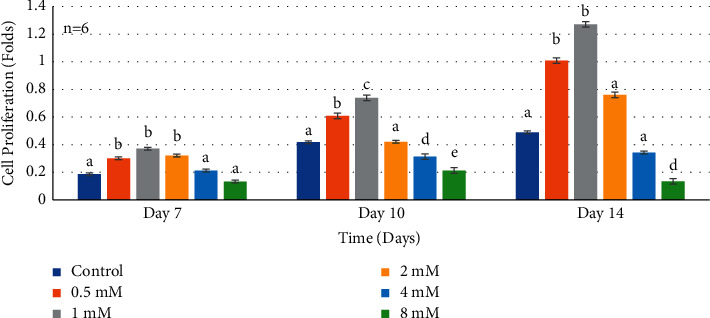
Histogram showing proliferation rates of normal human dental pulp cells in media with supplemental Mg^2+^ concentrations. Note: normal human dental pulp cells were cultured with media containing supplemental Mg^2+^concentrations 0.5 mM, 1 mM, 2 mM, 4 mM, and 8 mM and 0 mM as the control group after addition of preinductive dentinogenic media for time periods of 7, 10, and 14 days. The data are presented as means of six replicates with error bars indicating the standard deviation. Groups labeled with different letters differ statistically as compared to the control group and other study groups (*P* < 0.0001).

At 7 days, HDPCs proliferated to a significantly higher degree in supplemental Mg^2+^ concentrations 0.5 mM, 1 mM, and 2 mM compared to the negative control (*P* < 0.0001) (Figure 3). On the other hand, the remaining two higher Mg^2+^ concentrations 4 and 8 mM did not proliferate the rates at day 10 but rather behaved similar to the negative control. Higher Mg^2+^ concentrations resulted in a significant downregulation as shown in Figure 3. Wilks' lambda interaction *P* value for Mg^2+^ concentrations and their proliferation at this time interval showed a statistically significant value (*P* < 0.0001). At 10 days, Mg^2+^ concentrations 0.5 mM, 1 mM, and 2 mM showed significantly increased proliferation rates (Figure 3) (*P* < 0.0001). While, 4 mM and 8 mM Mg^2+^concentrations revealed proliferation rates of HDPCs similar to that of the negative control (Figure 3). Wilks' lambda interaction *P* value at this time interval presented statistically significant values (*P* < 0.0001). The data at day 14 presented an enhanced proliferation (Figure 3) in the 0.5 mM, 1 mM, and 2 mM Mg^2+^concentrations (*P* < 0.0001) However, higher Mg^2+^concentrations 4 mM and 8 mM showed decreased proliferation rates in comparison to the negative control, with Wilks' lambda *P* value at this time point showing a highly statistically significant value (*P* < 0.0001).

### 3.4. Effect of Mg^2+^ on Alkaline Phosphatase Activity of HDPCs at Various Concentrations

**Figure 4:**
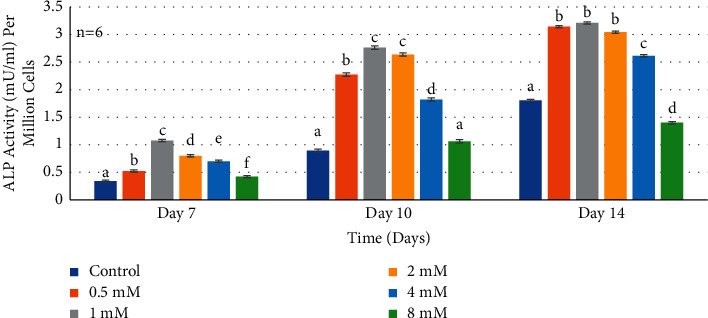
Histogram showing alkaline phosphatase activity of normal human dental pulp cells in media with supplemental Mg^2+^concentrations. Note: normal human dental pulp cells were cultured with media containing supplemental magnesium concentrations 0.5 mM, 1 mM, 2 mM, 4 mM, and 8 mM and 0 mM as the control group for time periods of 7, 10, and 14 days. Alkaline phosphatase activity in supernatants was normalized per million cells at each time interval. The control cells were treated with growth media without supplemental Mg^2+^. The data are presented as means of six replicates with error bars indicating the standard deviation. Groups labeled with different letters differ statistically as compared to the control group and other study groups (*P* < 0.0001).

Alkaline phosphatase activity observed at day 7 (Figure 4) for most groups was comparable to the negative control with a statistically significant difference. The Mg^2+^concentrations 0.5 mM, 1 mM, and 2 mM showed high statistically significant increase in ALP activity of HDPCs compared to the negative control (*P* < 0.0001). However, marked significant increase in enzymatic activity was noted with the 1 mM Mg^2+^concentration compared to the negative control and the other concentrations, with Wilks' lambda interaction *P* value for Mg^2+^concentrations and ALP activity at day 7 showing a statistically significant value (*P* < 0.0001). At day 10 (Figure 4), the 1 mM supplemental Mg^2+^ concentration reached a higher statistically significant value. However, cells grown in media containing 8 mM Mg^2+^supplement showed statistically lower ALP activity compared to the negative control. Wilks' lambda interaction *P* value for Mg^2+^concentrations and ALP activity at the 10th day timepoint showed a marked significant value (*P* < 0.0001). At day 14 (Figure 4), the 1 mM supplemented Mg^2+^concentration reached the highest value compared to the negative control. Data revealed that Mg^2+^concentrations in the range 0.5–2 mM have the highest effect on ALP activity. Meanwhile, cells grown in media containing higher Mg^2+^supplements (8 mM) showed lower ALP activity compared to the negative control. Wilks' lambda interaction *P* value for Mg^2+^concentrations and ALP activity at the 14th day timepoint showed the same statistically significant value (*P* < 0.0001).

### 3.5. Effect of Mg^2+^ on Mineralization Rate of HDPCs at Various Concentrations

**Figure 5:**
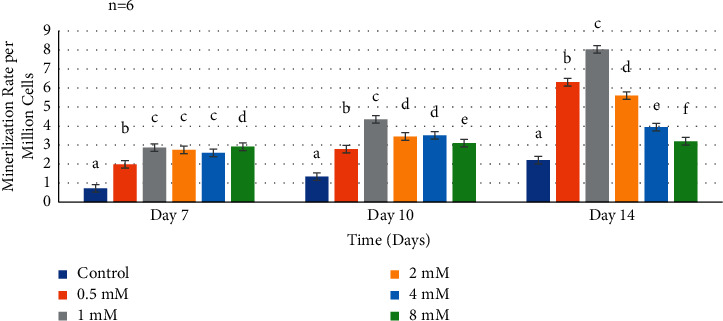
Mineralization rate of human dental pulp cells in media with supplemental Mg^2+^ concentrations. Normal human dental pulp cells were cultured with media containing supplemental Mg^2+^concentrations 0.5 mM, 1 mM, 2 mM, 4 mM, and 8 mM and 0 mM as the control group for time periods of 7, 10, and 14 days. Mineralization of fixed cell samples was measured by spectroscopic analysis at 405 nm and normalized per million cells at each time interval. The control cells were treated with growth media without supplemental Mg^2+^. The data are presented as means of six replicates with error bars indicating the standard deviation. Groups labeled with different letters differ statistically as compared to the control group and other study groups (*P* < 0.0001).

At day 7 (Figure 5), most Mg^2+^concentrations showed a statistically significant increase in the mineralization rate comparable to the negative control (*P* < 0.0001). Wilks' lambda interaction *P* value for Mg^2+^ concentrations and mineralization rate at day 7 presented a value of a highly statistical significance (*P* < 0.0001). However, at day 10, data from the Mg^2+^ concentrations 0.5–2 mM presented higher mineralization rates when compared to the negative control (Figure 5). As for the 1 mM Mg^2+^ concentration, there was a marked increase in value compared to the other Mg^2+^ concentrations and the negative control (*P* < 0.0001). Wilks' lambda interaction *P* value for Mg^2+^ concentrations and mineralization rate at day 10 pointed out a marked significant value (*P* < 0.0001). Similarly, the increase in mineralization rate could also be observed at day 14 (Figure 5), as Mg^2+^ concentration groups ranging 0.5–2 mM showed higher mineralization comparable to the negative control and the other concentrations (*P* < 0.0001). While, the 4 mM and 8 mM Mg^2+^concentrations showed a decreased mineralization rate of a statistical significance compared to the other experimental groups (*P* < 0.0001). Regarding, Wilks' lambda interaction *P* value, for Mg^2+^ concentrations and mineralization rate at day 14, a markedly significant value was also noted (*P* < 0.0001).

## 4. Discussion

The vitality of the dental pulp has always been the goal for a successful long-term restorative dental treatment. In situations of deep cavitation or trauma that involves dental pulp exposure, a cascade of stem cell activation, proliferation, and differentiation into new odontoblast-like cells will culminate into reparative dentine secretion [37]. The response of the pulp to direct capping involves dentinogenesis resulting from the recruitment of odontoblasts and/or proliferation of undifferentiated mesenchymal cells [38]. By the creation of biological sealing for exposed dental pulp, the connection between dental pulp and the oral environment is eliminated, and the entry of pathogens into the dental pulp is prevented. It has been postulated that a network of interactions between extracellular matrix molecules [39, 40] and growth factors regulates odontoblast-like cell differentiation and reparative dentinogenesis in the dental pulp [41]. In spite of a wide research made in the field of pulp physiology, there is no single gold standard regimen for pulp capping materials that can achieve reliable and predictable goals preserving tooth vitality. The identification and development of appropriate biomaterials for dental pulp capping are necessary to optimize clinical approaches to dentinogenesis. Likewise, a better understanding of the interactions between the microenvironment, growth factors, and human dental pulp cells will provide design basis to fulfill this purpose. The introduction of bioactive Mg^2+^ ions might explore the potential to overcome challenges of dentinogenesis. Therefore, the aim of this present study was to test and determine the effects of different concentrations of supplemental Mg^2+^ on HDPCs in terms of cell attachment, proliferation, differentiation, and mineralization in order to establish an optimal concentration to develop a new pulp-capping material.

HDPCs are a promising cell source for dental tissue regeneration due to their ability to differentiate into odontoblast-like cells in vitro and to form dentin-pulp structures in vivo when seeded on scaffolds [42]. Most of the studies investigating the growth effects of Mg^2+^ were performed in cell lines, transformed cells, or highly passaged and induced pluripotent stem cells [43–48]. In this study, the explant outgrowth technique was used leading to the isolation of the HDPCs from pulp tissue on the basis of their morphological features including the fibroblast-like cells and the stellate types. For the present work, HDPCs were isolated from the pulp tissue of extracted human third molar teeth. Several studies revealed that osteogenic cells surrounding Mg-containing biomaterials showed enhanced osteogenic activities [49, 50]. It has been shown from in vitro cell line and human cell studies, as well as in vivo studies that additional Mg^2+^ increases bone density and bone absorption parameters [51–61]. It has been reported that the osteogenic phenotypes of osteosarcoma cell lines are significantly different from normal human osteoblast cell lines [49, 53, 59, 62–66]. Osteosarcoma cells proliferate significantly more rapidly than normal human osteoblast cells [67–69], and inversely, the differentiation rate is significantly greater in normal human osteoblasts than in any osteosarcoma cell lines tested [70–72]. Franks et al. [73] observed upregulation of cell proliferation on ATCC human osteosarcoma cell line MG-63 by increasing MgO concentration over 7 mol% in the bioglass system. In a study by Burmester et al. [74], 0.02 g/ml Mg-based extracts were used, and the results showed that the proliferation rates on the ATCC MG-63 and U20S cell lines were normal, but on ATCC human, Sa0S-2 cell line was downregulated. Moreover, Park et al. [75] found that Mg^2+^ and magnesium-hydroxyapatite (Mg-HA) coatings on titanium improved ALP activity in MC3T3-E1 cell line. Lü et al. [65] claimed that up to 70 mM extracellular supplemented Mg^2+^ originated from MgCl_2_ could upregulate proliferation of normal rabbit osteoblasts and reached the best result at 30 mM. Yang et al. [76] reported adult human bone marrow-derived stromal cells (hBMS) cultured in extracts of magnesium. The results indicated that 10 mm concentration of Mg^+2^ did not inhibit the viability and osteogenic differentiation of hBMS cells. Lu et al. [77] tested the effect of supplemented MgCl_2_ concentrations (0.5 mM–16 mM) on the osteogenic behaviors of normal human primary osteoblasts. They demonstrated the impact of 2 mM Mg^2+^on induced proliferation and differentiation of normal human osteoblasts. It is evident that Mg^2+^ has an influence on cell attachment, proliferation, and differentiation activity of osteoblasts, stimulating expression of growth factors and early osteogenic markers, inducing bone formation.

Incorporating Mg^2+^ ions into calcium phosphate cement (CPC) was reported previously. In vitro studies showed that the release of Mg^2+^ ions in CPC cement promoted the proliferation and differentiation of human bone marrow stem cells and enhanced the activity of osteoblast differentiation [78, 79]. One of such approaches was biomimetic nanostructured nanofibrous gelatin magnesium phosphate scaffolds (NF-gelatin/MgP) by Qu et al. [80]. This hybrid scaffold not only physically and chemically mimicked the dentin matrix but also provided sustained Mg ion release from the matrix, thus creating favorable physical and chemical cues to guide dental stem/progenitor cell growth and differentiation. In vitro and in vivo studies showed that the NF-gelatin/MgP hybrid scaffold significantly enhanced human DPSC proliferation, differentiation, and new dentin formation compared to the NF-gelatin control. The abovementioned studies emphasized that magnesium containing scaffolds are ideal tools for regenerative endodontic therapy, releasing high extracellular magnesium to enhance dentin regeneration in stem cells. However, to date, the exploration of Mg-pulp capping biomaterials for dental tissue regeneration has not been attempted. This study could be the first report on dentinogenic stimulation of HDPCs by the optimal Mg^2+^ concentration.

Mg^2+^ promotes cell adhesion via 5*β*1- and *β*1-integrin-associated signal transduction pathways, which are involved in the enhanced activation of the key signaling adaptor protein Shc (Src homology collagen), resulting in the gene expression of extracellular matrix proteins [81]. Surface chemistry modification with Mg^2+^ also plays an important role in focal adhesion kinase (FAK; pp125^FAK^) mediated signal transduction via cell-surface integrin-ECM interaction. It has been shown that FAK expression, collagen type 1, vitronectin, and fibronectin are enhanced in osteoblasts growing on Al_2_O_3_-Mg^2+^, suggesting that tyrosine phosphorylation of signaling proteins was enhanced by binding to Mg^2+^supplemented bioceramics [81]. These findings suggested that the supplemental Mg^2+^ ions might also deliver its effect on the attachment of HDPCs. In this study, supplemental Mg^2+^ was used in the following concentrations 0.5 mM, 1 mM, 2 mM, 4 mM, and 8 mM, with a control of 0 mM. The results showed that there was higher attachment when relatively optimal concentrations were used. Specifically, the highest attachment was noticed with Mg^2+^ in 0.5 mM and 1 mM compared to the control. However, higher concentrations (4 mM and 8 mM) of Mg^2+^ had no effect on attachment compared to the optimal concentrations of 0.5 mM and 1 mM. These results may be attributed to the chemical structure of Mg^2+^which triggers human dental pulp cell signaling pathways affecting cellular motility. In a study by Sana et al. [82], human dental pulp stem cells when placed in contact with chitosan scaffolds were not able to attach nor spread on the surface since chitosan lacks adhesion motifs. Lu et al. [77] evaluated the in vitro effect of Mg^2+^ (0.5–16 mM) on osteogenic phenotypic behaviors of normal human osteoblasts in terms of attachment efficiency. They concluded that no cell doubling occurred, and cell attachment efficiency was not significantly affected by any Mg^2+^ ion concentration when compared to the control. In contrast, the data of the present study showed the highest attachment in` HDPCs with 1 mM supplement Mg^2+^. In a study by Shimaya et al. [83], magnesium enhanced adherence of synovial MSCs through *α*3 and *β*1 integrins, which promoted synthesis of cartilage matrix. These results confirm with the present data demonstrating the essential role of Mg^2+^ in HDPCs adhesion.

Pioneering work by Rubin [84] implicated Mg^2+^ as a key factor of the so-called “coordinated control of cell proliferation.” Mg^2+^ is involved in DNA duplication and plays a role in cytoskeleton rearrangement leading to the formation of the mitotic spindle and cytokinesis [84]. At the subcellular level, Mg^2+^ regulates contractile proteins, modulates transmembrane transports of Ca^2+^, Na^+^, and K^+^, and controls metabolic regulation of the energy-dependent cytoplasmic and mitochondrial pathway [85]. Proliferating cells have more Mg^2+^ than nonproliferating cells and high extracellular Mg^2+^ [86]. Mg^2+^ has been shown to induce cell proliferation in mammalian cells, including neural cells, keratinocytes, endothelial cells, fibroblasts, and lymphocytes. In the present study, assessment of proliferation rate of HDPCs was upregulated as noticed during the entire experiment when the supplemented Mg^2+^ values ranged from 0.5 mM to 2 mM, compared to the control group. These results are in accordance with Montezano et al. [87] and Lu et al. [77] who similarly showed an upregulatory effect by 2 mM concentration of MgCl_2_. They reported induced cell cycle activation and increased DNA and protein synthesis in the proliferated vascular smooth muscle cells (VSMCs) and normal human osteoblasts, respectively. The concentration of Mg^2+^ that was focused upon in the aforementioned studies was 2 mmol/L, as this level falls within the physiological/pathophysiological range (normal serum Mg^2+^ 0.6–1.5 mmol/L), unlike other studies that used concentrations as high as 5–10 mmol/L, which have little physiological or pathological relevance. Whereas, higher Mg^2+^ concertation groups ≥4 mM showed a down regulatory effect on HDPCs proliferation rate. These results are in agreement with Lu et al. [77], Leidi et al. [88], and Kircelli et al. [89], who reported that higher Mg^2+^concentrations behaved similar to the control cells grown on culture media inhibiting the proliferation activity of normal human osteoblasts. Despite the fact of using normal human osteoblasts and human pulp cells, both cell types showed a similar behavior responding to the same optimal Mg^2+^ concentration groups and interestingly at the same time points. Higher Mg^2+^ concentrations in the culture medium might interfere with the ion balance in the plasma membrane, leading to cytotoxicity and therefore inhibiting cellular proliferation and differentiation activities [90].

Mg^2+^ can regulate its own homeostasis and, hence, its intervention in cell differentiation as an authentic cell regulator. During dentin formation, odontoblast cells synthesize and secrete noncollagenous proteins in the dentin extracellular matrix; among these, ALP plays a regulatory role in mineralization and osteo-dentin/reparative dentin and are considered specific markers for dentinogenic phenotype [91]. ALP is an early marker for dentinogenic differentiation. The activity of ALP might be a prerequisite for the differentiation of pulp cells into odontoblasts. Expression of ALP by cultured human dental pulp stem cells (hDPSCs) has been determined and used as a marker for the cells' ability to produce the mineralized matrix in vitro [92, 93]. Recent investigations have shed new light on the mechanism of ALP action in promoting mineralization [91, 94]. In addition, investigation of the key association of ALP with cell membranes and matrix vesicles and the complex interactions of lipids, proteins, and ions ultimately result in the nucleation and propagation of mineral crystals promises to reveal new insights into how cells utilize the unique properties of ALP to form mineral [91, 95]. The accelerated differentiation and biomineralization from the addition of Mg^2+^ ions were believed to be attributed to the activation of ALP and promotion of crystallization processes and pattern formation of the inorganic mineral phase [96]. The data of the present study revealed that at 7 days, optimal Mg ^2+^ concentration groups (0.5 mM, 1 mM, and 2 mM) showed an increase in the rate of ALP activity. Moreover, at days 10 and 14, the same concentration groups had a significantly higher increase in the rate of ALP activity. Notably, cells grown in media containing higher Mg^2+^supplements (8 mM) showed lower ALP activity compared to the control at 14 days. Comparing ALP activity at different time intervals throughout the experiment clearly demonstrated the direct stimulating effect of ALP activity with 0.5–2 mM supplemental Mg^2+^ concentrations on HDPCs as evidenced by increase in ALP expression among these groups. Results presented by Lu et al. [77] and Burmester et al.' [74] studies are in agreement with these experimental data in relation to the effect of MgCl_2_ on ALP activity of HDPCs. Contrastingly, the data presented by Yang et al. [76] and Leidi et al. [88] demonstrated that <10 mM concentration of Mg^2+^ did not inhibit the viability, osteogenic differentiation, and ALP activity of human bone marrow mesenchymal stem cells.

In the present study, adding optimal concentrations of supplemented Mg^2+^ ranging 0.5 mM–2 mM at time intervals of 7, 10, and 14 days resulted in a significantly increased effect of upregulating mineralization compared to the control group. Optimal Mg^2+^ concentrations could stimulate the expression of calcium deposition of HDPCs. However, higher Mg^2+^ concentrations 4 mM and 8 mM groups downregulated calcium deposition. In this study, it was noticed that the results of the assays of mineralization and ALP activity revealed a consistent pattern for the same concentrations at the same time points in HDPCs. These results coincide with those of Lu et al. [77], showing the same trend regarding the concentration range and the time points, but in different types of cells. Therefore, these findings confirm that both normal HDPCs and human osteoblast could exhibit similarities in biochemical responses to optimal Mg^2+^ supplement concentration.

Zhang et al. [97] tested the effect of extracellular Mg^2+^ (0.8–3.8 mM) on the deposition of calcium phosphate matrix and Ca^2+^ signaling on human bone marrow-derived mesenchymal stem cells (hBMSC). They reported that Mg^2+^ concentration over 1.3 mM suppressed mineralization of mesenchymal stem cells, which is in disagreement with our study where optimal Mg^2+^ concentrations (0.5–2 mM) groups had an upregulating effect on HDPCs, increasing mineralization and calcium deposition. The difference between these data could be attributed to the addition of calcium. Optimized Ca^2+^ is critical for ALPase activity [98], and therefore, although Mg^2+^ concentrations are within 0.5–1.2 mM, the physiologic range of freely available Mg^2+^ calcium concentrations could impact ALPase activity.

The effect of the pH on bone mineralization and repair has been previously reported [99–102]. On a cellular level, during metabolic acidosis, osteoblast functions declined, whereas during metabolic alkalosis, osteoblast functions, such as cell viability, ALP activity, and mineral deposition, increased [103–105]. The acid-base microenvironment formed by reaction of magnesium chloride with water produces magnesium cation and chloride anion (Mg^2+^ and Cl^−^) [106]. The microenvironment where the cells reside can be affected by ions arising from the material dissolution and by concomitant pH changes affecting cell behavior [103]. Studies have shown that an increment in Mg^2+^ concentration caused by its degradation forms a high osmotic pressure and that the alkaline environment caused by released hydroxide ions coinhibited cell proliferation [107]. However, some studies have revealed that the proliferation of rat bone marrow mesenchymal cells (rBMSCs) on Mg^2+^-doped TiN ((Ti, Mg) N) coatings is associated with the amount of released Mg^2+^. Nevertheless, none of previous studies was done to verify the effect of extracellular pH on human pulp cells. Understanding and controlling pertinent cell functions by modulating the local engineered extracellular environment, specifically pH, is certainly a critical issue in future studies, for developing suitable pulp capping materials for dental tissue regeneration.

## 5. Conclusions

The data of this study suggested a significant benefit imparted by Mg^2+^ supplements of 0.5–2 mM on HDPCs evidenced by upregulated attachment rate, proliferation, differentiation, alkaline phosphatase activity, and mineralization, leading to potential of an improved pulp-capping material. This is the first report to demonstrate the optimal Mg^2+^concentrations needed to significantly enhance the dentinogenic activities of normal HDPCs. Mg-containing biomaterials could be considered for a future novel dental pulp-capping material in regenerative endodontics including improving direct pulp-capping and pulpotomy procedures. Future in vitro studies would be needed to verify the biological effect of magnesium on reparative dentin formation.

## Data Availability

The data used to support the findings of this study are included within the article and are available from the corresponding author upon request. The raw data used to support the findings of this study are stored at the X220 Research Lab according to Boston University policy.
